# Changes in Particle Size, Sedimentation, and Protein Microstructure of Ultra-High-Temperature Skim Milk Considering Plasmin Concentration and Storage Temperature

**DOI:** 10.3390/molecules26082339

**Published:** 2021-04-17

**Authors:** So-Yul Yun, Jee-Young Imm

**Affiliations:** Department of Foods and Nutrition, Kookmin University, Seoul 02707, Korea; soyul9175@naver.com

**Keywords:** ultra-high-temperature (UHT) skim milk, plasmin, storage temperature, particle size, sedimentation, microstructure

## Abstract

Age gelation is a major quality defect in ultra-high-temperature (UHT) pasteurized milk during extended storage. Changes in plasmin (PL)-induced sedimentation were investigated during storage (23 °C and 37 °C, four weeks) of UHT skim milk treated with PL (2.5, 10, and 15 U/L). The increase in particle size and broadening of the particle size distribution of samples during storage were dependent on the PL concentration, storage period, and storage temperature. Sediment analysis indicated that elevated storage temperature accelerated protein sedimentation. The initial PL concentration was positively correlated with the amount of protein sediment in samples stored at 23 °C for four weeks (*r* = 0.615; *p <* 0.01), whereas this correlation was negative in samples stored at 37 °C for the same time (*r* = −0.358; *p <* 0.01) due to extensive proteolysis. SDS-PAGE revealed that whey proteins remained soluble over storage at 23 °C for four weeks, but they mostly disappeared from the soluble phase of PL-added samples after two weeks’ storage at 37 °C. Transmission electron micrographs of PL-containing UHT skim milk during storage at different temperatures supported the trend of sediment analysis well. Based on the Fourier transform infrared spectra of UHT skim milk stored at 23 °C for three weeks, PL-induced particle size enlargement was due to protein aggregation and the formation of intermolecular β-sheet structures, which contributed to casein destabilization, leading to sediment formation.

## 1. Introduction

Ultra-high-temperature (UHT) pasteurized milk combined with aseptic packaging extends the shelf life of milk by up to several months with minimal sensory changes and nutrient losses. The continuously increasing global market demand for UHT milk is due to its long shelf life and stability without refrigeration until consumption [1]. This trend of the UHT milk market is expected to continue increasing between 2020 and 2025 at an annual average growth rate of 5.34% [2].

Age gelation refers to the loss of fluidity of milk because of increased viscosity. Destabilization of casein (CN) micelles and subsequent protein aggregation results in the formation of an irreversible gel during extended storage. Age gelation deteriorates the quality of dairy products and usually occurs in concentrated milk products and long shelf-life UHT milk [3,4]. The mechanisms of age gelation are still not clear, but the release of heat-induced β-lactoglobulin (β-LG) and κ-CN complexes from CN micelle surfaces to serum has been suggested as the initiation of age gelation [5].

Plasmin (PL), an indigenous heat-stable milk protease, is actively involved in age gelation by liberating β-LG-κ-CN complexes from CN micelles. PL is primarily present as its inactive zymogen, plasminogen (PG). PG activators, PG activator inhibitors, and PL inhibitors modulate the total PL activity in milk [6]. Due to PL and PG displaying greater heat tolerance than their inhibitors, attributed to protection by CN, heat treatment can sometimes promote PL-induced proteolysis depending on the heating conditions [7]. The complete inactivation of PL and PG is not normally achieved in the UHT process, resulting in conversion from PG to PL during storage of UHT milk. One approach to improving the storage stability of UHT milk is to preheat the milk at 90 °C for 30–60 s prior to direct UHT, effectively inactivating PL [8]. Heat-induced disulfide bond formation between β-LG and PG contributes to the decreased PL activity [6].

PL-induced proteolysis also plays an important role in flavor and texture development during cheese ripening [9]. The addition of PL (>1 mg/mL) was found to result in decreased curd strength and cheese yield [10]. It has been reported that notable PL activity was maintained in various milk protein ingredients and that variations in the functionality of CN-based milk ingredients depend on the residual PL activity [11]. Although the mechanisms of age gelation and sedimentation of UHT milk have been critically reviewed [1,12], direct, experiment-based data regarding the extent of PL-induced proteolysis on the sedimentation of UHT milk during storage is fairly limited.

To enhance the understanding of sedimentation in UHT skim milk due to PL-induced proteolysis, we added various concentrations of PL to UHT skim milk and monitored the changes in particle size distribution, sediment formation, and microstructure formation during storage at two different temperatures (23 °C and 37 °C). In addition, changes in the secondary structures of skim milk proteins leading to increased sedimentation were analyzed by Fourier transform infrared (FT-IR) spectroscopy.

## 2. Results and Discussion

### 2.1. Changes in Particle Size Distribution of PL-Added UHT Skim Milk Stored at Different Temperatures

**Figure 1:**
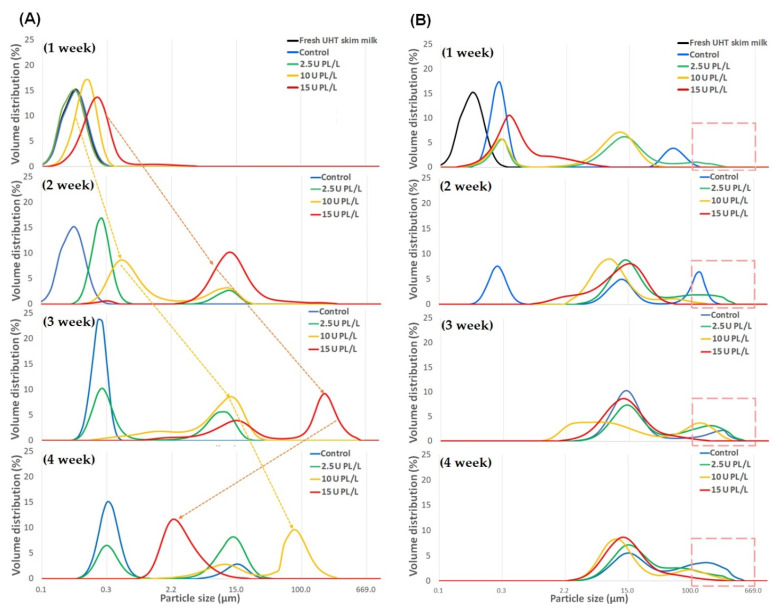
Changes in particle size distribution of UHT skim milk containing 2.5, 10, and 15 U PL/L during storage at (**A**) 23 °C and (**B**) 37 °C. UHT, ultra-high temperature; PL, plasmin.

The effects of PL addition and storage temperature on the particle size distribution of UHT skim milk were examined. Control skim milk (no PL addition) displayed a particle size distribution in the range of 0.02–0.4 μm, close to the typical average CN micelle size of 0.2 μm [13]. The increase in particle size and broadening of the particle size distribution of samples during storage were dependent on the PL concentration, storage period, and storage temperature. During storage at 23 °C, particles greater than 100 μm were not observed in any sample until two weeks. Particles greater than 100 μm were first observed in the samples containing 10 and 15 U PL/L at four (24%) and three weeks (54%) of storage, respectively (Figure 1A). The proportion of particles greater than 100 μm started decreasing in the UHT skim milk containing 15 U PL/L after storage for three weeks.

Storage at 37 °C caused more rapid changes in the particle size of samples than storage at 23 °C, probably due to increased PL activity at the higher storage temperature [1]. In UHT skim milk containing 15 U PL/L, the proportion of particles greater than 100 μm was lower than that of the control or skim milk containing 2.5 U PL/L throughout storage (Figure 1B). These results suggested that increased PL-mediated proteolytic activity hindered the formation of large-sized protein aggregates due to hydrolytic degradation of the CN molecules, facilitating their solubilization.

### 2.2. Changes in Protein Sedimentation Content of PL-Added UHT Skim Milk Stored at Different Temperatures

**Figure 2:**
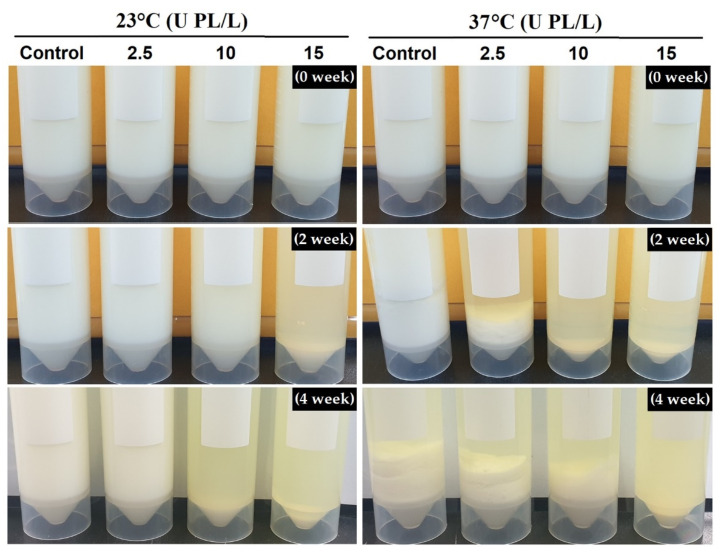
Changes in appearance of UHT skim milk containing 2.5, 10, and 15 U PL/L during storage. UHT, ultra-high temperature; PL, plasmin.

The UHT skim milk samples destabilized as storage period progressed due to the disintegration of the CN micelles. Distinct separation into one concentrated formation (sediment) and another depleted formation (transparent supernatant) of protein was visually observed. The time when the sediment first appeared varied, depending on the PL concentration and storage temperature. Sediment formation was first observed for samples stored for two weeks at 23 °C and 37 °C containing 15 U PL/L and ≥2.5 U PL/L, respectively (Figure 2). In other words, lower PL concentration was required to induce sedimentation at higher storage temperature.

Centrifugation of UHT skim milk resulted in phase concentration of the destabilized CN micelles. The amount of protein sediment was significantly affected by the added PL concentration and storage period (*p <* 0.01, Table 1). Overall, protein sediment was greater in the samples stored at 37 °C than at 23 °C for the same storage period. It showed that extensive PL-induced hydrolysis at elevated storage temperature (37 °C) promoted sediment formation rather than solubilization, as observed at 23 °C.

The amount of protein sediment in the control and UHT skim milk containing 2.5 U PL/L increased with storage at 23 °C, whereas it decreased in the samples containing 10 and 15 PL/L at four and three weeks, respectively. This decrease might be due to partial solubilization of protein fragments. For the samples stored at 37 °C, the amount of protein sediment increased continuously in the control and the samples containing 2.5 and 10 U PL/L. On the contrary, there was substantially less protein sediment in UHT skim milk containing 15 U PL/L at three and four weeks than in the other samples in the same storage periods.

Table 2 indicates the Pearson correlation coefficients between the added PL concentration, storage period, and protein sediment at the two different storage temperatures. The PL concentration and amount of protein sediment formed during storage were positively correlated at 23 °C (*r* = 0.615; *p <* 0.01) and negatively correlated at 37 °C (*r* = −0.358; *p <* 0.01). A higher correlation was obtained between the amount of protein sediment and storage period at 37 °C than at 23 °C (R = 0.768 vs. 0.361; *p <* 0.01). This result indicated the opposing effects of temperature and PL-induced proteolysis on sedimentation. Elevated storage temperature significantly promoted sedimentation, but extensive PL-induced proteolysis decreased sediment formation.

The far greater sediment observed at four weeks in control UHT skim milk stored at 37 °C than in skim milk containing 15 U PL/L stored at 23 °C suggested that PL-induced proteolysis was not the sole contributor to sediment formation and that other physicochemical parameters were involved in the destabilization of proteins at elevated storage temperature. In previous studies, high storage temperature shortened onset of gelation time regardless of the extent of proteolysis [14], and much less time was required to reach a similar sedimentation level in UHT milk stored at 30 °C and 40 °C than at 22 °C [15]. Anema demonstrated that reconstituted UHT skim milk formed non-enzymatic sediment and age gelation without notable proteolysis, concluding that aggregation of κ-CN-depleted CN micelles was responsible for age gelation [16]. Increased sedimentation is facilitated at elevated storage temperature because increased ionic calcium promotes aggregation of κ-CN depleted CN micelles [17]. In the present study the final pH levels after storage at 23 °C and 37 °C for four weeks were 6.60 and 6.45, respectively (data not shown). The relatively greater pH decline at 37 °C probably increased the solubilization of calcium and critically affected the stability of κ-CN-depleted CN micelles. Physicochemical changes leading to accelerated sedimentation of UHT milk at high storage temperature primarily involve changes in the secondary structure of proteins due to increased proteolysis and protein crosslinking through Maillard-type reactions [18,19]. In addition, PL-induced proteolysis in UHT milk can destabilize CN micelles by weakening CN–CN and CN–calcium phosphate interactions, essential for CN micelle integrity [20].

### 2.3. Changes in Proteolysis in PL-Added UHT Skim Milk Stored at Different Temperatures

**Figure 3:**
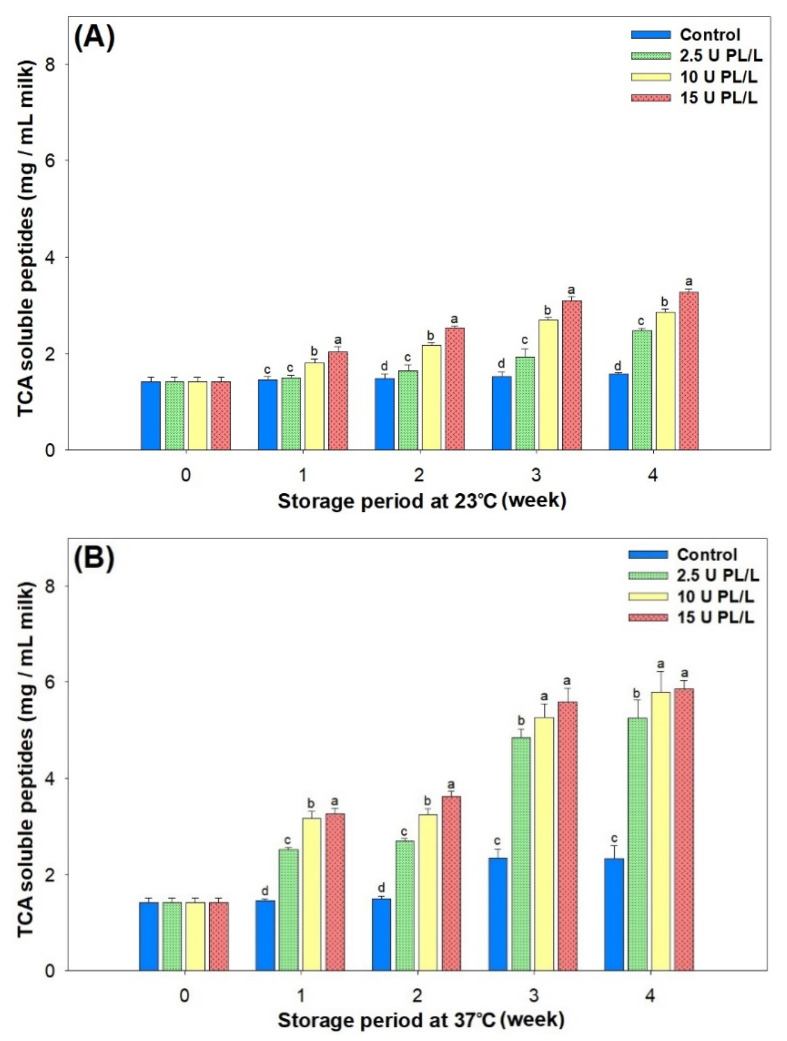
Changes in TCA soluble peptide content of UHT skim milk samples containing 2.5, 10, and 15 U PL/L during storage at (**A**) 23 °C and (**B**) 37 °C. ^a–d^ Different letters indicate significant difference within the same storage period (*p* < 0.05). TCA, trichloroacetic acid; UHT, ultra-high temperature; PL, plasmin.

Changes in TCA (12%, *w*/*v*) soluble peptides during storage were analyzed as an index of the extent of proteolysis. TCA soluble peptides increased significantly as the initial concentration of PL in the samples was increased and as storage progressed (*p <* 0.05, Figure 3A,B).

Proteolysis was accelerated during storage at elevated temperature (37 °C vs. 23 °C). The TCA soluble peptides in control skim milk increased by approximately 1.7-fold after storage at 37 °C for four weeks, indicating that significant residual PL activity remained even after UHT pasteurization. PL displays optimum activity at 37 °C around pH 7.4 and it hydrolyzes the C-terminal peptide bond of lysine and arginine residues with a preference for Lys-*X* bonds [21]. The sensitivity of CN molecules to PL increases in the order of β-CN = α_S2_-CN > α_S1_-CN > κ-CN [1]. However, major whey proteins, such as β-LG and α-lactalbumin, are not sensitive to PL because of their globular structure [22]. The sensitivity of κ-CN to PL is still uncertain, and its hydrolytic activity varies depending on the PL concentration [23]. Gaucher et al. [24] reported that κ-CN derived peptides were produced during storage of UHT milk both at 20 °C and at 40 °C, possibly due to κ-CN proteolysis by PL and other proteases from somatic cells.

Although autolysis-mediated decreased proteolysis was reported upon two weeks’ storage at 37 °C [25], it was not unequivocally observed in the present study, even in the control UHT skim milk. The extent of proteolysis reflected by TCA soluble peptides did not show a linear correlation with the amount of sediment formed during storage.

### 2.4. Protein Profiles of the Soluble Phase and Sediment in PL-Added UHT Skim Milk Stored at Different Temperatures

**Figure 4:**
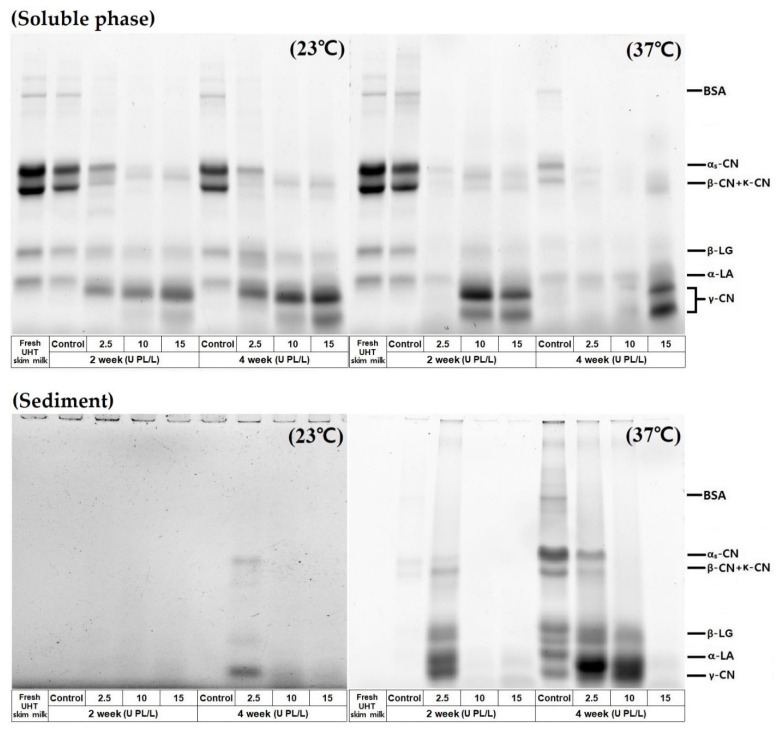
SDS-PAGE protein profiles of soluble and sediment phase of UHT skim milk containing 2.5, 10, and 15 U PL/L stored at 23 °C and 37 °C for up to four weeks. The sediment produced during storage was obtained by centrifugation (27,000× *g*) at 4 °C for 10 min and redispersed in distilled water by sonication. UHT, ultra-high temperature; PL, plasmin; BSA, bovine serum albumin; CN, casein; β-LG, β-lactoglobulin; α-LA, α-lactalbumin.

The protein profiles of the soluble and sediment phases were analyzed by SDS-PAGE. As shown in Figure 4, the rapid disappearance of α_S_-/β-CN and generation of β-CN products (γ-caseins) occurred in the soluble phase during storage at 37 °C. Interestingly, whey proteins bands remained in the soluble phase of all samples stored at 23 °C, even at four weeks, whereas they had mostly disappeared from the soluble phase of PL-added samples stored at 37 °C for two weeks. This result supports the finding that denatured whey proteins contribute to the stabilization of CN micelles by inhibiting CN aggregation [17].

The protein composition of the sediment was not well-visualized in the samples stored at 23 °C because of the low protein content. In samples stored at 37 °C, protein compositions of the sediment varied depending on the PL concentration. Significant amounts of intact α_S_-/κ-CN, and β-LG appeared in the sediment of the control skim milk at four weeks of storage. This implies that whey proteins in the soluble phase reacted with soluble protein aggregates or PL-induced β-CN derived peptides (γ-CNs) and eventually co-sedimented when the size of the protein aggregates reached the limit of solubilization. Extensive hydrolysis of α_S_-CN was not required for sediment formation. Extensive CN hydrolysis continued in the samples containing high PL concentrations and produced sediment consisting of low molecular weight CN fragments. These hydrolyzed protein fragments possibly suppressed the formation of large protein aggregates. The protein composition changes in the soluble phase and sediment phase suggest that the addition of external PL and storage temperature affect aggregation and rearrangement of the supermolecular structure formation via modulation of the extent of proteolysis and the medium condition. Holland et al. [26] reported that storage rather than processing displayed a more critical effect on protein crosslinking in UHT milk and this manifested at elevated storage temperatures, such as 40 °C.

### 2.5. Changes in Microstructure of PL-Added UHT Skim Milk Storored at Different Temperatures

**Figure 5:**
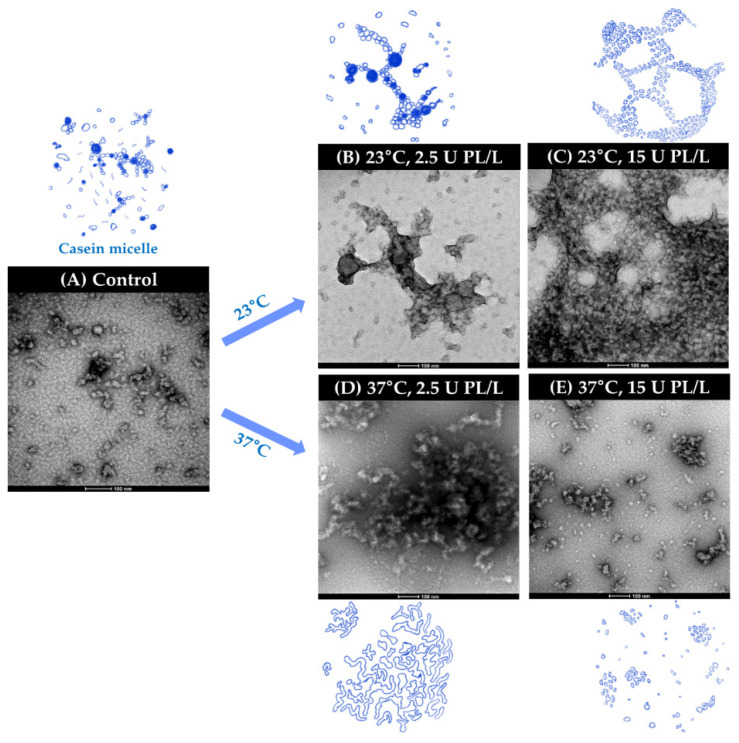
TEM images of PL-added UHT skim milk at different storage conditions. (**A**) Control (fresh UHT skim milk); (**B**) UHT skim milk containing 2.5 U PL/L stored for three weeks at 23 °C; (**C**) UHT skim milk containing 15 U PL/L stored for three weeks at 23 °C; (**D**) UHT skim milk containing 2.5 U PL/L stored for three weeks at 37 °C; (**E**) UHT skim milk containing 15 U PL/L stored for three weeks at 37 °C. UHT, ultra-high temperature; PL, plasmin.

The microstructure of PL-added UHT skim milk at different storage temperatures was analyzed by TEM, and representative images were recorded. As shown in Figure 5A, CN micelles were observed in the form of particles and clusters or chains.

The protein network of UHT skim milk containing 2.5 U PL/L became less fine and presented an aggregated form after storage at 23 °C for three weeks (Figure 5B). The protein network formation was most dense in the sample containing 15 U PL/L and stored at 23 °C (Figure 5C). In the samples stored at 37 °C for three weeks, the reinforcement of protein chains and clusters was observed, with fiber-like protein bridges appearing in the skim milk containing 2.5 U PL/L (Figure 5D). On the contrary, extensive proteolytic breakdown of the protein network was noticed in the sample containing 15 U PL/L after storage for three weeks (Figure 5E). Raynes et al. [27] reported that heating a mixture of κ-CN and β-LG produced unique amyloid-like co-aggregates which differed from structures produced by the individual proteins. The PL-induced liberation of κ-CN-β-LG complexes from CN micelles might act as a bridge to form fibril-like fibrous structures, as shown in Figure 5B,C.

Based on these results, proteolysis is a major contributor to the destabilization and subsequent aggregation of milk proteins. The aggregated protein structure formation varies depending on the storage temperature and PL activity. The aggregated protein network, which was closely related to sediment formation, increased even with a low PL concentration (2.5 U PL/L) at 37 °C. The small peptides generated by extensive hydrolysis increased the solubilization of proteins and inhibited protein–protein interactions, resulting in no network formation. Although Malmgren et al. [15] could not provide direct evidence for changes in protein network structures, they showed decreased gelation of UHT milk stored at 40 °C. The rapid PL-induced proteolysis at high temperature also decreased physical contact and associations between proteins, thereby decreased protein gelation. TEM images of PL containing UHT skim milk during storage at different temperatures supported the sediment analysis results well (Table 1).

### 2.6. PL Concentration-Dependent Secondary Structure Changes in UHT Skim Milk during Storage

Possible PL-mediated changes to protein structure leading to sedimentation were analyzed by FT-IR. The samples stored at 23 °C for three weeks were used for this analysis because of their distinct PL concentration-dependent particle size changes. The secondary structures of the samples were calculated using amide I (1600~1700 cm^−1^) and amide II (1500~1600 cm^−1^) spectral bands by the Gaussian function fitting method. As shown in Table 3, the most noticeable change in the secondary structures of the proteins in UHT skim milk was the increased intermolecular β-sheet structure. The proportion of intermolecular β-sheet structures in skim milk containing 15 U PL/L was increased by 1.9-fold while turns decreased by 0.67-fold compared to those of the control after storage at 23 °C for three weeks.

This result suggests that the breakdown of α_S_- and β-CN to smaller peptide fragments during PL-induced proteolysis resulted in conformational changes affecting the secondary structure conversion. Overall, the degree of structural changes was related to the PL concentration (or extent of proteolysis), and degradation of PL-sensitive β-CN and α_S1_-CN possibly accelerated structural conversion. These changes, in turn, impaired the chaperone ability of CN molecules, which prevented amorphous protein aggregation and rendered CN network formation [28]. Increased exposure of hydrophobic regions in proteins induces self-association and results in sedimentation of amorphous aggregates [29].

Taken together, PL-induced particle size enlargement was due to protein aggregation, and the formation of intermolecular β-sheets structures contributed to CN destabilization, leading to sedimentation. Similar to the result of the present study, sediment formation in UHT milk during storage was positively correlated with increased intermolecular β-sheet structures [30]. In addition, protein cross-linking and aggregate formation involving Maillard-type reactions cannot be ruled out in during the storage of UHT milk because of the possible generation of reactive amino groups and increased lactose–lysine interactions in skim milk containing high PL activity.

## 3. Materials and Methods

### 3.1. Preparation of UHT Skim Milk Containing Various Concentrations of PL

Freshly-produced UHT skim milk was purchased from a local supermarket (Seoul, Korea). Bovine PL (Sigma-Aldrich, St. Louis, MO, USA) was added to UHT skim milk at 2.5, 10, and 15 U/L. Sodium azide (0.02%, *w*/*v*) and bronopol (0.0005%, *w*/*v*) were also added to prevent undesirable microbial growth. The addition of PL and preservatives to samples was done on a clean bench. UHT skim milk samples containing PL at various concentrations were stored either in a 23 °C or a 37 °C incubator for up to four weeks, respectively.

### 3.2. Particle Size Distribution

Changes in the particle size distribution of samples during storage were measured using a particle size analyzer (Horiba LA-960 Laser Scattering Particle Size Analyzer, Kyoto, Japan). Samples were diluted to appropriate transmittance (85–97%), and the volume particle size distribution of samples was measured in triplicate.

### 3.3. Sediment Analysis

The sediment produced during storage was obtained by centrifugation at 27,000× *g* at 4 °C for 10 min (Avanti JE, Beckman, Fullerton, CA, USA). After the sediment was redispersed in distilled water using an ultrasonic device (JAC 4020, KODO, Seoul, Korea), the protein content was quantified with the bicinchoninic acid (BCA) assay [31].

### 3.4. Protein Profiles of the Soluble Phase and Sediment

The protein profiles of the soluble phase and sediment recovered from sediment analysis (two and four weeks) were analyzed by SDS-PAGE. Aliquots of samples (10 μL) were loaded onto a precast polyacrylamide gradient gel (Any kD^TM^, Bio-Rad Laboratories, Richmond, CA, USA) and separated in a Bio-Rad mini gel electrophoresis unit at 40 V. The protein bands were visualized using the Bio-Rad ChemiDoc XRS + system.

### 3.5. Extent of Proteolysis

The amount of trichloroacetic acid (TCA, Sigma-Aldrich, St. Louis, MO, USA; 12%, *w*/*w*) soluble peptides was used as an index of the extent of proteolysis. Each sample was mixed with an equal volume of TCA (24%, *w*/*v*) and left undisturbed for 10 min before centrifugation (14,000× *g*, 4 °C for 30 min). Soluble peptides in the supernatant were quantified in triplicate using the BCA assay.

### 3.6. Transmission Eectron Microscopy (TEM)

TEM images were obtained using the method of Raynes et al. [27] with slight modifications. Samples (5 μL) were mounted on the carbon-coated 300-mesh copper grids with a hydrophilic surface and were adsorbed for 60 s. After staining with aqueous uranyl acetate (2%, *w*/*v*), the samples were examined under a transmission electron microscope (TEM, Talos L120C, FEI, Hillsboro, OR, USA) at an operating voltage of 120 kV. The representative images of samples were recorded at 45,000× magnifications.

### 3.7. FT-IR

The samples stored at 23 °C for three weeks were used to estimate the PL-induced structural changes in the skim milk proteins because these samples displayed detectable PL concentration-dependent particle size changes compared to samples stored at 37 °C, which presented substantial protein degradation and difficulty in identifying the structural changes leading to protein aggregation. The changes in global secondary structures of skim milk proteins were analyzed by the FT-IR method of Qi et al. [32] with slight modification. Freeze-dried samples (1 mg) were mixed with potassium bromide (KBr, 100 mg; PIKE Technologies, Madison, WI, USA) and pressed into thin pellets under high pressure (14,000 psi). The samples were placed on the cell holder of a PerkinElmer Frontier FT-IR Spectrometer (Vertex 70, Bruker Optics, Ettlingen, Germany) and scanned at 20 °C from 4000 to 600 cm^−1^ with a resolution of 2 cm^−1^ and 128 accumulative scans. The peak fit procedure (Origin 9.0 software, OriginLab, Northampton, MA, USA) was used to separate major peaks. Each peak was identified and fitted with a Gaussian function. Secondary structure was classified into intermolecular β-sheet (1694–1682 cm^−1^/1620–1610 cm^−1^), turns (1682–1662 cm^−1^), α-helix (1660–1650 cm^−1^), disordered (1650–1640 cm^−1^), and intramolecular β-sheet (1640–1620 cm^−1^) [33]. The heights of all the assigned structural element component peaks were summed and divided by the total integrated height of peaks to obtain the percentage of the element.

### 3.8. Statistical Analysis

All quantitative determinations were performed in triplicate and expressed as means ± standard deviation. Two-way analysis of variance (ANOVA) was used to examine the effect of added PL concentration and storage period on the amounts of protein sedimentation and TCA-soluble peptides. When data showed significant differences (*p* < 0.05), Tukey’s post hoc test was used for multiple comparisons among treatment means. Pearson correlation coefficient analyses were used to examine the relationship between the added PL concentration and storage period at the two different temperatures. All statistical analyses were performed using SPSS Statistics V. 21 (SPSS Inc., Chicago, IL, USA).

## 4. Conclusions

A PL-added UHT skim milk model system was chosen to accelerate sedimentation, mimicking the age gelation observed during prolonged storage. The increased proteolysis accelerated sedimentation, but the rapid protein breakdown reduced protein aggregation and subsequent sediment formation of destabilized micelles. The early sediment formation in UHT skim milk containing high PL activity suggests that PL-induced destabilization occurred during storage. PL-induced proteolysis was not the sole contributor to sediment formation, and other physicochemical parameters were involved in destabilization of proteins at elevated storage temperature. PL-mediated proteolysis decreased the chaperone activity of CN and promoted the formation of intermolecular β-sheet structures, leading to sedimentation during storage. Future studies identifying the conditions required to strengthen the chaperone action of CN by modification of heating regimes and serum environments might help reduce the occurrence of age gelation in UHT milk, prolonging its shelf life.

## Figures and Tables

**Table 1:** Protein sediment analysis of UHT skim milk containing 2.5, 10, and 15 U PL/L.

Storage Period (Week, 23 °C)	Protein Sediment (mg)				Storage Period (Week, 37 °C)	Protein Sediment (mg)				
	Added PL Concentration (U PL/L)					Added PL Concentration (U PL/L)				
	Control	2.5	10	15		Control	2.5	10	15	
0	0.31 ± 0.01 ^Az^	0.31 ± 0.01 ^Az^	0.31 ± 0.01 ^Az^	0.31 ± 0.01 ^Az^	0	0.31 ± 0.01 ^Az^	0.31 ± 0.01 ^Az^	0.31 ± 0.01 ^Az^	0.31 ± 0.01 ^Az^	
1	0.43 ± 0.02 ^Cy^	0.48 ± 0.01 ^Cy^	6.39 ± 0.12 ^By^	7.17 ± 0.17 ^Aw^	1	0.69 ± 0.01 ^Cz^	21.25 ± 0.36 ^Ay^	6.48 ± 0.48 ^By^	5.90 ± 0.09 ^By^	
2	0.58 ± 0.01 ^Dx^	1.02 ± 0.01 ^Cx^	6.66 ± 0.20 ^By^	7.69 ± 0.18 ^Bv^	2	5.01 ± 0.10 ^Dy^	49.39 ± 0.23 ^Ax^	8.22 ± 0.13 ^Bx^	7.66 ± 0.25 ^Cx^	
3	0.53 ± 0.06 ^Dx^	3.00 ± 0.11 ^Cw^	9.43 ± 0.20 ^Aw^	5.19 ± 0.11 ^Cx^	3	70.13 ± 0.25 ^Bx^	71.05 ± 0.45 ^Aw^	47.13 ± 0.22 ^Cw^	11.68 ± 0.19 ^Dw^	
4	0.60 ± 0.02 ^Dx^	3.83 ± 0.03 ^Bv^	8.14 ± 0.03 ^Ax^	2.98 ± 0.08 ^By^	4	93.45 ± 1.99 ^Aw^	95.50 ± 0.59 ^Av^	80.55 ± 0.42 ^Bv^	14.05 ± 0.36 ^Cv^	
Two-way ANOVA analysis	Significance				Two-way ANOVA analysis	Significance			
Interactions of storage time × added PL concentration	**				Interactions of storage time × added PL concentration	**			

Data were expressed as mean ± standard deviation (n = 3). Data were subjected to statistical analysis using two-way ANOVA. Different capital superscripts in the same column or different small superscripts in the same row at designated storage temperatures indicate significant difference at *p <* 0.05. Significance: ** *p <* 0.01.

**Table 2:** Pearson correlation coefficients for the relationship between added PL concentration, storage period, and protein sediment.

	Protein Sediment	
	23 °C	37 °C
Added PL concentration	0.615 **	−0.358 **
Storage period	0.361 **	0.768 **

** *p <* 0.01, PL, plasmin.

**Table 3:** Secondary structure analysis of amide I region obtained by Fourier-transform infrared spectroscopy from UHT skim milk stored at 23 °C for three weeks.

Samples	Intermolecular β-Sheet (%)	Turns (%)	α-Helix (%)	Disordered (%)	Intramolecular β-Sheet (%)
Control	15	30	18	23	15
2.5 U PL/L	19	20	22	24	15
10 U PL/L	21	21	22	22	14
15 U PL/L	28	20	15	22	15

The ranges of each secondary structure were assigned as follows [16]: intermolecular β-sheet (1694–1682 cm^−1^/1620–1610 cm^−1^), turns (1682–1662 cm^−1^), α-helix (1660–1650 cm^−1^), disordered (1650–1640 cm^−1^), and intramolecular β-sheet (1640–1620 cm^−1^). PL, plasmin.

## Data Availability

The data presented in this study are available on the request from the corresponding author.

## Sample Availability

Samples of the compounds are not available from authors.
